# Bioactivity-Guided High Performance Counter-Current Chromatography and Following Semi-Preparative Liquid Chromatography Method for Rapid Isolation of Anti-Inflammatory Lignins from Dai Medicinal Plant, *Zanthoxylum acanthopodium* var. *timbor*

**DOI:** 10.3390/molecules28062592

**Published:** 2023-03-13

**Authors:** Qing-Fei Fan, Lan Zhou, Pian-Chou Gongpan, Chuan-Li Lu, Hua Chang, Xun Xiang

**Affiliations:** 1 College of Science, Yunnan Agricultural University, Kunming 650201, China; 2 CAS Key Laboratory of Tropical Plant Resources and Sustainable Use, Xishuangbanna Tropical Botanical Garden, Chinese Academy of Sciences, Xishuangbanna 666303, China; 3 College of Food and Drug Engineering, Guangxi Vocational University of Agriculture, Nanning 530007, China; 4 Institute of Bioengineering, Guangdong Academy of Sciences, Guangzhou 510316, China; 5 College of Vetezrinary Medicine, Yunnan Agricultural University, Kunming 650201, China; 6 College of Animal Science and Technology, Yunnan agricultural University, Kunming 650201, China

**Keywords:** *Zanthoxylum acanthopodium* var. *timbor*, anti-inflammatory activity screening, high-performance countercurrent chromatography, preparative separation

## Abstract

The development of Dai medicine is relatively slow, and *Zanthoxylum* has great economic and medicinal value. It is still difficult to obtain medicinal components from the low-polarity parts of *Zanthoxylum* belonging to Dai medicine. In this study, we introduced one simple and quick strategy of separating target compounds from the barks of *Z. acanthopodium* var. *timbor* by high-performance countercurrent chromatography (HPCCC) with an off-line anti-inflammatory activity screening mode. The development of this strategy was based on the TLC-based generally useful estimation of solvent systems (GUESS) method and HPCCC in combination. This paper presented a rapid method for obtaining target anti-inflammatory compounds. Three lignins were enriched by HPCCC with an off-line inhibition mode of nitric oxide production in lipopolysaccharide-stimulated RAW264.7 macrophage cells, using petroleum ether–ethyl acetate–methanol–water (3:2:3:2) as the solvent system. The results showed that this method was simple and practical and could be applied to trace the anti-inflammatory components of the low-polarity part in Dai medicine.

## 1. Introduction

Dai medicine is one of the medicine systems of China’s four major ethnic groups (Tibetan, Mongolian, Uygur and Dai) and is also one of the ancient traditional medicines in China. Xishuangbanna is located at the intersection of the warm and wet air currents of the East Asian monsoon of the Pacific Ocean and the Southwest Indian Ocean monsoon. Dai people are prone to various diseases in such a humid location, and the Dai people have accumulated rich experience in traditional medicine in the long process of fighting against diseases [1,2].

At present, compared with other ethnic medicines, the development of Dai medicines is relatively slow, and it mainly focuses on the arrangement of prescriptions. The technical bottleneck as to why Dai medicine cannot be widely used or developed and utilized in modern medicine is that their pharmacological chemical components and mechanisms are not clear [3]. There are many problems that need to be solved in the development of Dai medicine. The first scientific method is to strengthen the systematic study of Dai medicine [4] and clarify its pharmacodynamic material basis [3].

The development and utilization of active ingredients in ethno-medicine has become a hot research topic in the present era and has broad prospects [3]. There are many kinds of active ingredients in Dai medicine, and they are characterized by high impurity, low content, complex structure, volatilization, degeneration and inactivation, which increase the difficulty of the separation of the active ingredients. At present, there are not many methods for the separation and purification of the active ingredients in Dai medicine. The traditional methods commonly used include column chromatography (silica gel column and gel column), macroporous adsorption resin, supercritical fluid extraction, membrane separation, high-performance liquid chromatography, medium-pressure chromatography and other technical methods. This is similar to the research of traditional Chinese medicine. Column chromatography is more comprehensive and systematic in separation and purification. Although column chromatography is simple to operate, it requires repeated separation operations and consumes a lot of time. Moreover, solid materials filled in the column will cause irreversible adsorption and contamination of the sample, and even denaturation and inactivation of the sample. It is difficult to separate high-purity products with a macroporous adsorption resin and medium-pressure preparation chromatography, which is suitable for the segmentation of crude extracts [5]. Supercritical fluid extraction is only suitable for the separation and purification of low-polar or non-polar substances and cannot be used for the separation of large polar compounds, so the application scope is relatively limited [6]. High-purity products can be obtained by high-performance liquid chromatography (HPLC), but the sample size is small, and the materials and equipment are relatively expensive, with a high cost, and difficult to realize for industrialization.

Countercurrent chromatography is derived from multipole discontinuous extraction. The original basic model for high-speed countercurrent chromatography (HSCCC) was invented by Dr. Yiochiro Ito, a Japanese American scholar, in 1966. In 1982, Dr. Ito developed a new technique for continuous and efficient liquid–liquid partition chromatography, which is known today as HSCCC. Countercurrent chromatography could effectively avoid irreversible adsorption, a loss of activity and the denaturation of samples due to its unique chromatographic characteristics [7,8]. Moreover, countercurrent chromatography could be applied to separate compounds with different polarity [9]. Therefore, it is suitable for the separation of the active components of Dai medicine [10]. In the past, much progress on bioactive-guided separation and the identification of natural products by countercurrent chromatography combined with other chromatography techniques or biotechnology was reported [11,12,13,14,15,16]. High-performance countercurrent chromatography (HPCCC) has more advantages compared with conventional countercurrent chromatography (CCC), such as more than 90% liquid stationary phase retention and higher flow rates. It could improve mass processed and time throughput [17,18]. 

*Zanthoxylum* plants are evergreen or deciduous trees, shrubs or woody vines, belonging to dioecious plants, there are about 250 species in the world, and they are distributed in tropical, subtropical and temperate zones. There are 39 species and 14 varieties in China, most of which are homologous to medicine and food [19]. Lan Zhou, one of our authors, reviewed the types and bioactivity of the compounds isolated from *Zanthoxylum* from 2015 to 2021 in her doctoral dissertation [20]. The chemical compositions of *Zanthoxylum* plants were reported, mainly involving alkaloids, lignans, flavonoids, terpenoids and so on [21,22,23]. *Zanthoxylum* plants have a variety of biological activities, such as anticancer, antibacterial, anti-inflammatory and so on [24,25,26,27].

*Zanthoxylum acanthopodium* var. *timbor*, also called ‘gou hua jiao’, is a varietas of *Z. acanthopodium*, which is distributed in the Guangxi, Yunnan, Sichuan and Tibet provinces of China. The tender leaves, roots and fruits of *Z. acanthopodium* var. *timbor* are mainly used by Dai people for chronic hepatitis, appendicitis, nephritis, rheumatic bone pain and stomachache [19]. There are few literature reports about the barks of *Z. acanthopodium* var. *timbor.*

In our previous study, the barks of *Z. acanthopodium* var. *timbor* have been proven to have anti-inflammatory properties. The petroleum ether extract (40 μg/mL) was found to have the potential of inhibiting the nitric oxide production in a mouse mononuclear macrophage cell (RAW 264.7). Therefore, some novel anti-inflammatory components may exist. The petroleum ether extract of the *Zanthoxylum* plant has special characteristics, such as a light yellow oil and strong adhesion. Therefore, it is difficult to separate and study this part by traditional column chromatography. In the present study, a method for the rapid separation of the target compounds from the barks of *Z. acanthopodium* var. *timbor* by HPCCC and the screening mode for the inhibition of nitric oxide (NO) was developed. Three target compounds were isolated rapidly, which were fargesin, epieudesmin and (-)-pluviatylol-4’-O-γ,γ-dimethylallyl ether. The anti-inflammatory activity and the potential toxicity of the two major compounds, fargesin and epieudesmin, on the production of tumor necrosis factor α (TNF-α) and interleukin 1β (IL-1β) in lipopolysaccharide (LPS)-induced THP-1 cells were studied. The establishment of this method would provide certain guiding significance for accelerating the research of Dai medicine.

## 2. Results and Discussion

### 2.1. Nitric Oxide (NO) Production Inhibitor Screening Experiment

General inflammation is the body’s normal immune response. However, once the regulation is unbalanced, cytokine storms will appear in the body’s tissues or organs, which will eventually produce acute and chronic inflammation and related inflammatory diseases. In severe cases, it will threaten people’s lives. Balancing and regulating the secretion of inflammatory mediators and cytokines in the immune system is one of the ways to produce anti-inflammation activity. Nitric oxide (NO) is one of the key denominators in inflammation and carcinogenesis. In a previous study, many samples and isolated compounds were evaluated for the anti-inflammatory activity by inhibiting the nitric oxide (NO) production in the lipopolysaccharide (LPS)-induced RAW 264.7 cells.

In order to verify the anti-inflammatory activity in vitro of the barks of *Z. acanthopodium* var. *timbor*, LPS-stimulated RAW 264.7 cells were screened for inhibitory activity against NO. The total extract and four extracts were screened. The results (Table 1) showed that the ethyl acetate and petroleum ether extracts of *Z. acanthopodium* var. *timbor* possessed a more than 70% inhibition rate of NO production compared with the model group at a concentration of 40 μg/mL, and the total extract also effectively inhibited the production of NO, while the inhibition activity of the n-butanol extract was relatively weak. From Table 1, some novel anti-inflammatory components were believed to exist in the petroleum ether extract, so we planned to isolate the compounds of the petroleum ether extract with basic anti-inflammatory activities. However, the petroleum ether extract appeared oily and light yellow and had strong adhesion, and it is difficult to study the extract part with conventional chromatographic separation.

### 2.2. Optimization of HPCCC Solvent Systems

The selection and optimization of the solvent system for CCC is the key to achieving the ideal separation effect of samples. Because there is no fixed solvent system mode for each kind of separation, only some methods could be used to select a suitable solvent system. Therefore, the selection and optimization of the solvent system for CCC is a major difficulty in the separation work, which takes up most of the time of the whole separation work [28,29]. Many factors should be considered during the process; the *K_D_* value of the targeted compounds is the key factor in HPCCC separation [7,30]. An ideal *K_D_* value of the targeted compounds is from 0.5–2 for CCC to 0.25–4 for HPCCC. Therefore, a suitable solvent system for CCC separation is not easy to find. Many countercurrent chromatography experts, including Dr. Yiochiro Ito, have given some good advice. The literature method is the most simple and fastest method to find the solvent system of high countercurrent chromatography. We could find the examples of similar substances according to the class of substances to be separated, and then adjust the solvent system of the literature according to the specific situation and carry out the experiment. In addition, thin-layer chromatography (TLC) and high-performance liquid chromatography (HPLC) are commonly used in the selection and optimization of the solvent system for CCC. The difference in the content and distribution coefficient of each component in the sample could be observed by observing the spot size and chromaticity in TLC, so as to select the solvent system. HPLC could accurately analyze the partition coefficient (K) of multiple compounds simultaneously to determine the solvent system. The application of the above methods requires a certain amount of experience, and it is difficult for beginners to master and independently operate countercurrent chromatography in a short time.

Dr. Guido F. Pauli’s team, which is from the Department of Medicinal Chemistry and Pharmacognosy, College of Pharmacy, University of Illinois, in Chicago, United States, developed a new method based on previous research about CCC. They could use thin-layer chromatography (TLC) to quickly predict suitable solvent systems for CCC. According to the TLC-based generally useful estimation of solvent systems (GUESS) method, when the *Rf* value of the target compound is near 0.5, the *K_D_* value of the compound would go into the range 0.25–2.5 [7]. The TLC-based GUESS method has been successfully applied to many cases [29,30,31]. The polarity of the petroleum ether extract of *Z. acanthopodium* var. *timbor* is relatively low, and n-hexane -ethyl acetate-methanol-water solvent systems were preferred. In practice, n-hexane is often replaced by petroleum ether in order to save on experimental costs. In our experiment, four TLC solvent systems composed of petroleum ether–ethyl acetate–methanol–water for HPCCC were prepared (Table 2). The solvents with different volume ratios of petroleum ether/ethyl acetate (4:1), (5:2), (1:1) and (3:2) were tested, and the Rf value of the target compound with petroleum ether/ ethyl acetate (3:2) could reach near 0.5. Finally, according to rules of the TLC-based GUESS method, petroleum ether–ethyl acetate–methanol–water (3:2:3:2) was chosen for HPCCC.

### 2.3. Screening of Anti-Inflammatory Activities of Different Fractions from HPCCC

**Figure 1:**
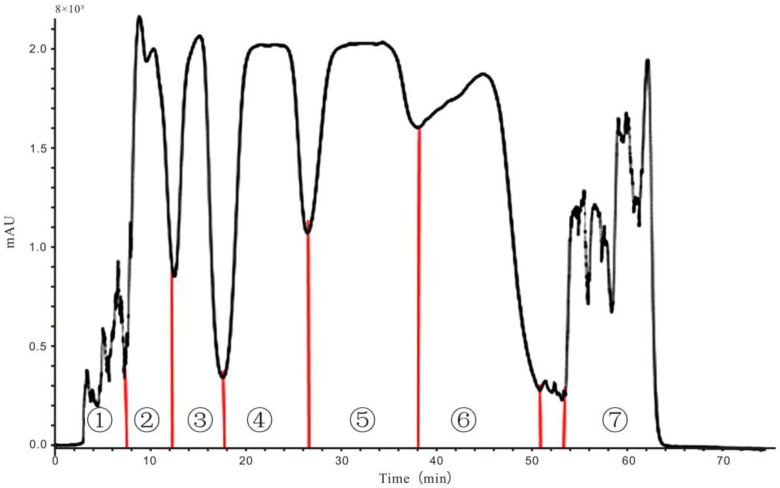
Chromatogram of one-step preparative HPCCC separation of the petroleum ether fraction of *Z. acanthopodium* var. *timbor.* Solvent system: n-hexane–ethyl acetate–methanol–water (3:2:3:2) with retention percentage of the stationary phase of 90%; stationary phase: upper phase; mobile phase: lower phase; resolution speed: 1600 rpm. UV detection wavelength: 210 nm. Fraction numbers were marked corresponding to the analysis result of the TLC chromatogram.

Petroleum ether extract possessed higher anti-inflammatory activity than other solvent extracts of *Z. acanthopodium* var. *timbor*. Therefore, the petroleum ether extract was further separated by HPCCC, yielding seven fractions Fr1–Fr7 (Figure 1). HPCCC could improve processing efficiency and save time compared with conventional CCC. We could quickly separate the relevant samples by HPCCC within an hour, while conventional CCC takes 12–24 h. Petroleum ether/ethyl acetate/methanol/water (3:2:3:2) was applied in the isolation in reversed phase mode with elution-extrusion mode. The upper phase was used as the stationary phase. The retention of the stationary phase was 90%. As shown in Figure 3, two peaks were obtained with Fr4 (89.9 mg) and Fr5 (99.8 mg), and corresponded to compounds **2** and **3**. The purities were about 90%, as determined by HPLC (Figure 3).

**Figure 2:**
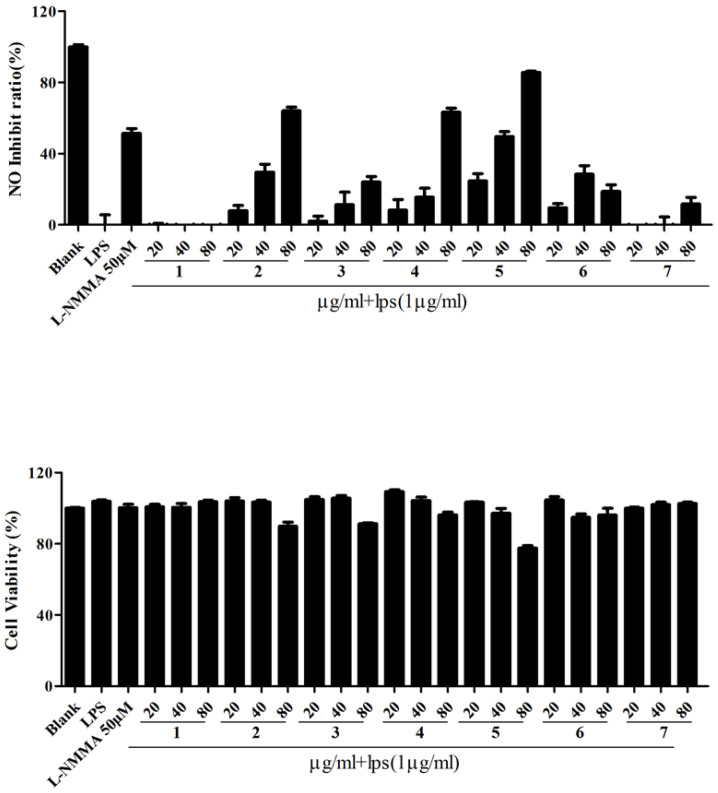
The inhibitions of fractions 1–7 from *Z. acanthopodium* var. *timbor* on the NO production in RAW264.7 cells induced by LPS. Note: all results are the average value after three repetitions plus minus standard deviation.

The anti-inflammatory bioactivities of these fractions were screened by inhibiting the NO production. Fr2, Fr4 and Fr5 inhibited the NO production in a dose-dependent manner in the concentration range of 20–80 μg/mL, while Fr2, Fr4 and Fr5 had little cytotoxicity with the concentration of 80 μg/mL (Figure 2). It was an interesting phenomenon. When the doses reached 40 μg/mL, the NO inhibition rates of Fr2 and Fr5 were close to the positive control and had no cytotoxicity. When the doses reached 80 μg/mL, the NO inhibition rates of Fr2, Fr4 and Fr5 were over 50%. Compared to the positive drug, the NO inhibition rates of Fr2 and Fr4 reached nearly 80% and Fr5 was close to 100%. At this point, Fr2 and Fr5 showed some cytotoxicity. Thus, we could draw a conclusion that Fr2, 4 and 5 possess significant anti-inflammatory activity. The cytotoxicity of the target compound needs more attention in future activity studies.

### 2.4. Purification of the Target HPCCC Peaks

**Figure 3:**
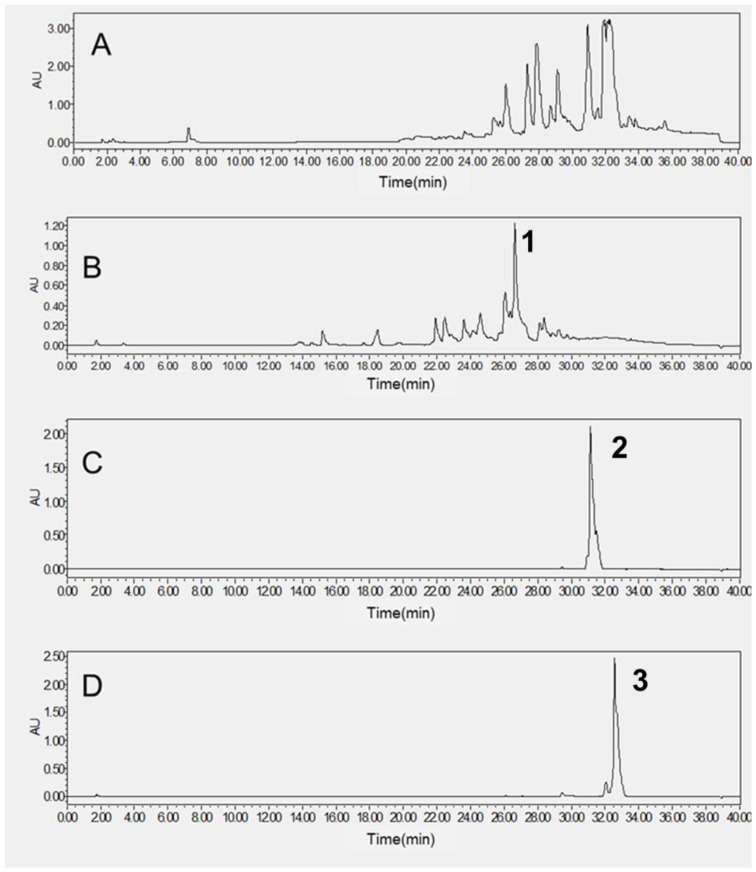
HPLC chromatogram of HPCCC peak of petroleum ether extract (**A**) and fractions Fr2 (**B**), Fr4 (**C**) and Fr5 (**D**).

Fr2, Fr4 and Fr5 were analyzed by HPLC using our patterned method (Figure 3). The purity of Fr2 was about 50%, while the purity of Fr4 and Fr5 was about 90%. Though there were many peaks in the HPLC diagram of Fr2, one major peak could be found in it (Figure 3B). Fractions 2, 4 and 5 were further purified by semi-preparative liquid chromatography before the structural identification and further investigation of the pharmacological actions. Fargesin (**1**) [32], epieudesmin (**2**) [18] and (-)-pluviatylol-4′-O-γ,γ-dimethylallyl ether (**3**) [33] were identified by the NMR data compared with the reported literature. These compounds are all lignins and isolated for the first time using this method.

Note: HPLC conditions: stationary phase, mobile phase, A (methonal)-B (water); gradient mode: 0–30 min, 5%A–95%A; 30–35 min, 95% A; 35–35.05 min, 95%A–5%A; 35.05–40 min. Flow rate: 0.8 mL/min. UV detection wavelength: 210 nm.

Compound **1**, white powder, ^1^H-NMR (500 MHz, CDCl_3_) *δ*: 6.93 (1H, s, H-2), 6.89–6.80 (4H, m, H-2′,5,6,6′), 6.78 (1H, d, *J* = 7.9 Hz, H-5′), 5.95 (2H, q, *J* = 1.5 Hz, -OCH_2_O-), 4.87 (1H, d, *J* = 5.7 Hz, H-7′), 4.42 (1H, d, *J* = 7.1 Hz, H-7), 4.12 (1H, d, *J* = 9.5 Hz, H-9b), 3.91 (3H, s, -OCH_3_), 3.88 (3H, s, -OCH_3_), 3.84 (2H, dt, *J* = 7.5, 6.0 Hz, H-9a, 9′b), 3.32 (2H, ddd, *J* = 16.8, 11.6, 7.9 Hz, H-8′, 9′a), 2.92–2.80 (1H, m, H-8). ^13^C-NMR (125 MHz, CDCl_3_) *δ*: 148.83 (C-3), 147.99 (C-4), 147.97 (C-3′), 147.21 (C-4′), 135.15 (C-1′), 130.92 (C-1), 119.59 (C-6′), 117.70 (C-6), 110.99 (C-5), 108.90 (C-2), 108.17 (C-5′), 106.56 (C-2′), 101.07 (-OCH_2_O-), 87.69 (C-7′), 82.03 (C-7), 71.00 (C-9′), 69.77 (C-9), 55.94 (3-OCH_3_), 55.91 (4-OCH_3_), 54.63 (C-8′), 50.16 (C-8).

Compound **2**, white powder, ^1^H-NMR (500 MHz, CDCl_3_) *δ*: 6.95–6.83 (6H, m, H-2, 2′, 5, 5′, 6, 6′), 4.88 (1H, d, *J* = 5.7 Hz, H-7), 4.45 (1H, d, *J* = 7.2 Hz, H-7′), 4.14 (1H, d, *J* = 9.5 Hz, H-9b), 3.91 (3H, s, -OCH_3_), 3.90 (3H, s, -OCH_3_), 3.89 (3H, s, -OCH_3_), 3.88 (3H, s, -OCH_3_), 3.87–3.83 (2H, m, H-9a and 9′a), 3.34 (2H, ddd, *J* = 16.8, 14.3, 8.1 Hz, H-8′ and 9′b), 2.92 (1H, dd, *J* = 14.7, 6.7 Hz, H-8). ^13^C-NMR (125 MHz, CDCl_3_) *δ*: 149.22 (C-4), 148.83 (C-4′), 148.72 (C-3), 148.00 (C-3′), 133.63 (C-1), 130.94 (C-1′), 118.50 (C-6), 117.71 (C-6′), 111.00 (C-5), 110.96 (C-5′), 109.08 (C-2), 108.92 (C-2′), 87.66 (C-7), 82.06 (C-′), 71.01 (C-9), 69.77 (C-9′), 55.96 (4′-OCH_3_), 55.94 (4-OCH_3_), 55.91 (3-OCH_3_, 3′-OCH_3_), 54.52 (C-8), 50.17 (C-8′).

Compound **3**, white powder, ^1^H NMR (500 MHz, CDCl_3_) *δ*: 6.92 (1H, d, *J* = 1.9 Hz, H-2′), 6.87 (1H, m, H-2), 6.85(1H, s, H-5′), 6.83(1H, d, *J* = 1.7 Hz, H-6), 6.82 (1H, d, *J* = 1.7 Hz, H-6′), 6.78 (1H, *s*, H-5), 5.95 (2H, *s*, H-10′), 5.53 (1H, t, *J* = 6.7 Hz, H-11), 4.85 (1H, d, *J* = 5.3 Hz, H-7), 4.58 (2H, d, *J* = 6.8 Hz, H-10), 4.42 (1H, d, *J* = 7.5 Hz, H-7′), 4.11 (1H, d, *J* = 9.4 Hz, H-9), 3.88 (3H, *s*, OCH_3_-3), 3.82 (2H, *m*, H-9 and 9′), 3.32 (2H, *m*, H-8 and 8′), 2.87 (1H, *m*, H-8),1.77 (3H, *s*, H-13), 1.73 (3H, *s*, H-14). ^13^C NMR (125 MHz, CDCl_3_) *δ*: 149.33 (C-4′), 147.96 (C-4), 147.27 (C-3), 147.20 (C-3′), 137.58 (C-12), 135.19 (C-1), 130.95 (C-1′), 120.03 (C-11), 119.58 (C-6), 117.61(C-6′), 112.96 (C-5′), 109.13 (C-2′), 108.16 (C-5), 106.56 (C-2), 101.06(-O-CH_2_-O-), 87.68 (C-7), 82.09 (C-7′), 71.00 (C-9), 69.79 (C-9′), 65.82 (C-10), 55.95 (3-OMe), 54.64 (C-8), 50.16 (C-8′), 25.86 (C-13), 18.24 (C-14).

### 2.5. The Anti-Inflammatory Activity of Fargesin (***1***) and Epieudesmin (***2***) on TPH-1 Cells

**Figure 4:**
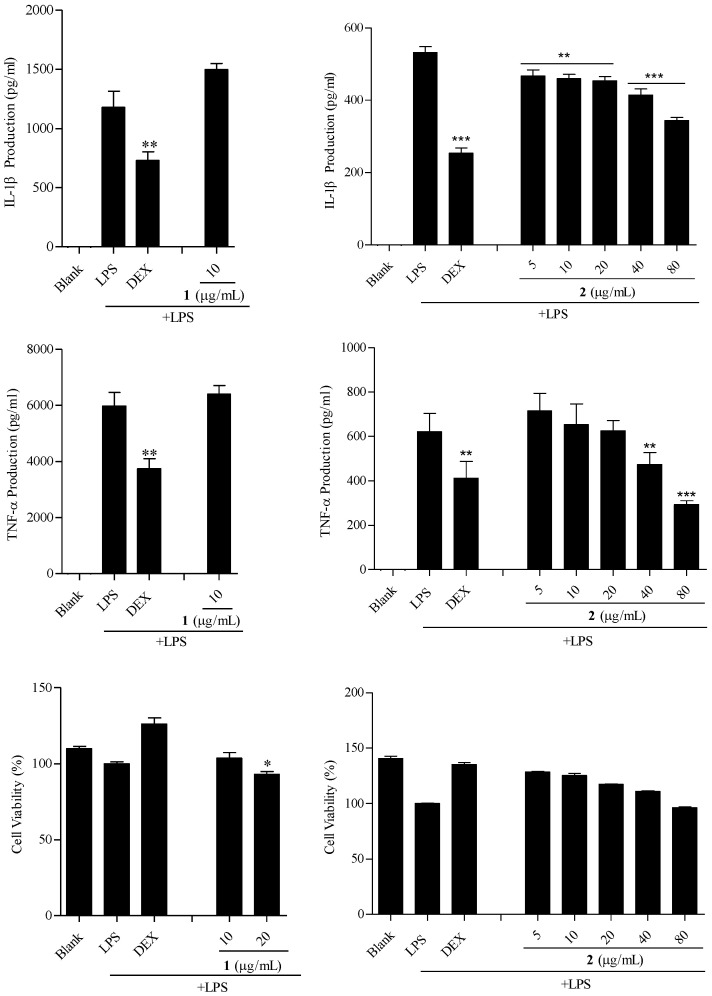
Inhibition of two compounds from *Z. acanthopodium* var. *timbor* barks on TNF-α and IL-1β production in LPS-stimulated THP-1 cells. Note: compound **1**: fargesin; compound **2**: epieudesmin. All results are the mean ± standard deviation after three repetitions. (* *p* < 0.05, ** *p* < 0.01, *** *p* < 0.001). *** *p* < 0.001 means that compared with the cells after LPS treatment, the sample has extremely significant inhibition on the production of IL-1β and TNF-α.

THP-1 cells are immune cells. Two major compounds, fargesin (**1**) and epieudesmin (**2**), isolated from *Z. acanthopodium* var. *timbor* on the production of tumor necrosis factor α (TNF-α) and interleukin 1β (IL-1β) in LPS-induced THP-1 cells was studied in the experiment (Figure 4). The results showed that compound **1** at 20 μg/mL has a certain cytotoxicity to THP-1 cells, and compound **2** not only has no cytotoxicity to THP-1 cells but also inhibits the TNF-α production on LPS-induced THP-1 cells at IC_50_ 361.79 ± 61.98 μmol/L. So, the experimental results showed that compound **2** has potential anti-inflammatory activity in vitro.

## 3. Materials and Methods

### 3.1. General Experimental Procedures

All NMR spectra were acquired on a Bruker Avance III 500 spectrometer, recorded in *δ* (ppm) using tetramethylsilane (TMS) as the internal standard. The coupling constants (*J*) were given in Hertz. Compound purification was performed in the Waters semi-preparative high-performance liquid chromatography system, which consisted of 1525 double pumps and 2487 detectors, and was equipped with a YMC-pack ODS-A column (250 × 10 mm, YMC Co., Ltd., Kyoto, Japan). Fractions were monitored by thin-layer chromatography (TLC) by silica gel GF254 (50 × 100 mm, Qingdao Marine Chemical Factory, Qingdao, China), and spots were visualized by heating silica gel plates and spraying with 10% H_2_SO_4_ in ethanol.

All chemical solvents used in this study were of analytical grade and purchased from Kunming Renke (Kunming, China) and Kunming Fuhaida (Kunming, China). The deionized water was disposed by Synergy UV Ultrapure water apparatus (Millipore, France).

The mouse mononuclear macrophage cells (RAW 264.7) and human monocytic cells (THP-1) were provided by Shanghai cell bank of Chinese Academy of Sciences (Shanghai, China). Lipopolysaccharide (LPS), Griess reagent (Reagent A and Reagent B, respectively), L-NMMA was purchased from Sigma (Shanghai, China). DMEM (high sugar) medium, RPMI 1640 medium, penicillin-streptomycin and fetal bovine serum (FBS) were obtained from Thermo Scientific (Shanghai, China). MTS reagent was purchased from Promega (Madison, WI, USA).

### 3.2. Plant Material

Dry stem barks of *Z. acanthopodium* var. *timbor* were collected on the high mountain of Majie Town, Yiliang County, Kunming City, Yunnan Province, in May 2017. The botanical authentication was carried out by the professor Dr. Qishi Song, Xishuangbanna Tropical Botanical Garden, Chinese Academy of Sciences. A voucher specimen (no.20170901) was deposited in the Laboratory of Ethno-medicine Research Group, Xishuangbanna Tropical Botanical Garden, Chinese Academy of Sciences.

### 3.3. Sample Preparation

The dried and shattered stem barks (10 kg) of *Z. acanthopodium* var. *timbor* were refluxed triple with 95% methanol. After concentration under reduced pressure, the 95% methanol extract was dissolved with water, and then extracted with petroleum ether, ethyl acetate and n-butanol four times, respectively. Four extracts were obtained and were divided into petroleum ether extract (392 g), ethyl acetate extract (329 g), n-butanol extract (250 g) and residual water phase fraction after solvent recovery under reduced pressure. The petroleum ether extract residue (500 mg) was loaded into HPCCC and eluted with petroleum ether /ethyl acetate/methanol/water (3:2:3:2) to produce seven fractions (1–7) based on TLC analysis. Fr1 (15.5 mg), Fr2 (34.4 mg), Fr3 (49.0 mg), Fr4 (89.9 mg), Fr5 (99.8 mg), Fr6 (52.6 mg) and Fr7 (60.1 mg) were obtained and stored in a refrigerator.

### 3.4. Selection of Two-Phase Solvent System

For CCC, selection of two-phase solvent system is the key process. Many factors should be considered in the process; *K_D_* value of the targeted compounds is the key factor in HPCCC separation [7,34]. TLC-based GUESS method represents a useful and practical method, which could link TLC retention factors (Rf values) and *K_D_* values in CCC. Based on previous studies, the optimized composition of the two-phase solvent system was carried by TLC-based GUESS method and analytical model of HPCCC [30,31]. According to the polarity of sample, different solvent systems are screened. n-hexane -ethyl acetate-methanol-water solvent systems were preferred for petroleum ether extract. In practice, n-hexane is often replaced by petroleum ether. The first step is to select the target compound, which is generally a large compound in crude extract. Solvents with different volume ratios of petroleum ether/ethyl acetate (4:1), (5:2), (1:1) and (3:2) were tested, and the Rf value of the target compound was monitored by TLC by silica gel GF254. Volume ratio of petroleum ether/ethyl acetate petroleum ether/ ethyl acetate (3:2) could move the Rf value of the target compound near 0.5. Finally, petroleum ether–ethyl acetate–methanol–water (3:2:3:2) was chosen for HPCCC.

### 3.5. HPCCC Separation Process

A Spectrum HPCCC instrument (Dynamic Extractions, Berkshire, UK) was used in the separation process and equipped with a quaternary gradient pump (KNAUER, Inc., Berlin, Germany). A Shanghaihuxi automatic sampling instrument BS-100N (Shanghai, China) was used for distillate collection. Petroleum ether/ethyl acetate/methanol/water (3:2:3:2) was applied in the isolation in reversed-phase mode, used at 25 °C. For the HPCCC separation, the stationary phase (upper phase) was first filled in the semi-preparative columns (total 300 mL) with a flow rate of 15 mL/min. The bobbins of the HPCCC equipment were rotating at 1600 rpm until the instrument was equilibrated in 5 min. Then, the mobile phase (lower phase) was pumped into the system using a flow rate of 15 mL/min until a hydrodynamic equilibrium was established. A total of 500 mg of the sample (dissolved in 6 mL of upper phase/lower phase, 1:1, *v*/*v*) was injected after the establishment of hydrodynamic equilibrium. Elution–extrusion mode was applied in the separation process. The elution run time was 20 min, and the extrusion time was 25 min at the same flow rate. The fractions were collected in 20 mL test tubes per minute. Finally, thin-layer chromatography was used to analyze each fraction and similar components were combined.

### 3.6. HPLC Conditions

HPLC analyses of samples were performed by an analytical Waters e2695 HPLC C18 column (5 μm, 4.6 × 250 mm) with an Extend C18-guard column. The HPLC separation was performed by using a linear gradient elution. The HPLC mobile phase composed of A (methanol) and B (water) at a flow rate of 0.8 mL/min. The gradient was used as follows: 0.00–30.00 min: 5%A–95%A; 30.00–35.00 min, 95%A; 35.00–35.05 min, 95%A–5%A; 35.05–40.00 min. The injection volume was 10 μL and the selected wavelength was 210 nm. The column temperature was set at 25 °C. 

### 3.7. The Screening of NO Generation Inhibitors

Murine macrophage cell line RAW264.7 cells were inoculated into 96-well plates. Except the blank control group, the other groups were stimulated with 1 μg/mL LPS. At the same time, the tested group and the positive control group were treated with 1–5 mg of methanol extract, petroleum ether extract, ethyl acetate extract and n-butanol extract of *Z. acanthopodium* var. *timbor* bark (final concentration of 40 μg/mL) and L-NMMA, respectively. After being cultured overnight, the production of NO was measured by Griess, which includes the absorbance value of NO detected by a microplate reader at 570 nm. MTS was added to the remaining medium to detect the cell viability [35,36].

The fractions from CCC were treated with serial dilutions with a concentration range of 20, 40, 80 μg/mL in triplicate, followed by stimulation with 1 μg/mL LPS for 18 h. NO production in the supernatant was assessed by Griess reagents. The absorbance at 570 nm was measured with a microplate reader. N^G^-Methyl-L-arginine acetate salt, a well-known nitric oxide synthase (NOS) inhibitor, was used as a positive control. The viability of RAW264.7 cells was evaluated by the MTS assay simultaneously to exclude the interference of the cytotoxicity of the test samples. 

Cell viability was measured by MTS assay as previously reported [35,36]. In MTS assay, 100 μL cell suspensions (1 × 10^6^ cells/mL) were cultivated in 96-cell plates for 18 h. Then, cells were pretreated with series concentrations of the four extracts and 10 μM/L DEX for 30 min before they were further incubated in the presence of 1 μg/mL LPS for 24 h. Finally, 20 μL of CellTiter 96^®^ A_Queous_ One Solution Reagent, prepared by MTS (3-[4,5,dimethylthiazol-2-yl]-5-[3-carboxymethoxy-phenyl]-2-[4-sulfophenyl]-2H-tetrazolium, inner salt) in the presence of phenazine ethosulfate (PES), was added to each well and incubated for 1 h at 37 °C in the 5% CO_2_ incubator. The absorbance of each well was measured at 490 nm directly using a Multifunctional Microplate Reader (Thermo Scientific VarioSkan Flash, Sunnyvale, CA, USA). The untreated cells, incubated in culture medium with 0.4% DMSO, were employed as blank controls in every experiment. Results were expressed as a percentage of untreated control cells. Values were presented as mean ± standard error of mean (SEM) of three independent tests.

### 3.8. Anti-Inflammatory Activity on THP-1 Cells

The anti-inflammatory activity in vitro was assessed on THP-1 cells. In all experiments, cells were pretreated with drugs about 30 min before LPS (1 μg/mL) stimulation. The production of pro-inflammatory cytokines (TNF-α and IL-1β) was tested by ELISA assay. All assays were performed in triplicate and dexamethasone (Dex, 10 mmol/L) was used as a positive control. Cell viability was measured by MTS assay as similar as part 3.7.

## 4. Conclusions

The pharmacodynamic components of a natural product could be divided into three parts according to their polarity. Natural products with a low polarity are mainly volatile oil, terpenoids and some flavonoids, among which volatile oil and diterpenoids have been studied more [37,38]. These low-polarity components are often found to have better biological or pharmacological activity in studies. It is difficult to control the quality and separation of natural products with a low polarity. In our previous experiment, it was found that the petroleum ether extract of the *Zanthoxylum* plant has special characteristics, such as a light yellow oil and strong adhesion. However, the low-polarity part is more active than the other parts. In urgent need of new drug research, it is necessary to introduce new chromatography technology to solve the above problems. Countercurrent chromatography, developed from liquid–liquid extraction, has the advantages of liquid–liquid extraction, which is different from conventional chromatography systems, and is very conducive to dealing with these problems. Countercurrent chromatography could effectively avoid irreversible adsorption, a loss of activity and the denaturation of samples due to its unique chromatographic characteristics. Therefore, it is suitable for the separation of the active components of Dai medicine. The TLC-based GUESS method is a useful method for the optimization of CCC solvent systems. The TLC-based GUESS method and HPCCC were employed for screening target compounds from the extracts of the barks. In this case, the target anti-inflammatory components were quickly enriched and separated by HPCCC and purified by HPLC. Therefore, the results showed that this method was simple and practical and could be applied to trace the anti-inflammatory components with low polarity in Dai medicine. The establishment of this method would provide certain guiding significance for accelerating the research of Dai medicine.

## Figures and Tables

**Table 1:** NO production inhibition rate of the sample.

Sample Name	Concentration	Inhibition Rate of NO Production (%)
L-NMMA	50 μM	51.88 ± 1.17
Residual water fraction	40 μg/mL	-
N-butanol extract	40 μg/mL	8.83 ± 1.67
Ethyl acetate extract	40 μg/mL	74.59 ± 1.40
Petroleum ether extract	40 μg/mL	93.88 ± 0.67
Total extract	40 μg/mL	39.35 ± 0.42

**Table 2:** Optimization of solvent systems.

NO	Solvent Systems (*v*/*v*/*v*/*v*) Petroleum Ether/Ethyl Acetate/Methanol/Water	Solvent Systems (*v*/*v*) Petroleum Ether / Ethyl Acetateof TLC	Average Rf Value
1	4:1:4:1	4:1	0.3
2	5:2:5:2	5:2	0.4
3	1:1:1:1	1:1	0.6
4	3:2:3:2	3:2	0.5

## Data Availability

All the data in this research were presented in the manuscript and Supplementary Material.

## Supplementary Materials

The following supporting information can be downloaded at: https://www.mdpi.com/article/10.3390/molecules28062592/s1, Figures S1–S6: One-dimensional NMR of compounds **1**–**3**.

## Sample Availability

Samples of compounds **1**–**3** are available from the authors.
