# Supplementary material for: Bioactivity-Guided High Performance Counter-Current Chromatography and Following Semi-Preparative Liquid Chromatography Method for Rapid Isolation of Anti-Inflammatory Lignins from Dai Medicinal Plant, Zanthoxylum acanthopodium var. timbor
Source: Molecules. 2023 Mar 13;28(6):2592. doi: 10.3390/molecules28062592 (PMC10057586; doi:10.3390/molecules28062592)
Supplement: Supplementary file 1 [file molecules-28-02592-s001.zip › molecules-2249556-supplementary.pdf]

molecules

MDPI

Article

Bioactivity-guided High Performance Counter-current Chromatography and Following Semi-preparative Liquid Chromatography Method for Rapid Isolation of Anti-inflammatory Lignins from Dai Medicinal Plant, *Zanthoxylum acanthopodium* var. *timbor*

Qing-Fei Fan [1,2,*,†], Lan Zhou [2,3,†], Pian-Chou Gongpan [2], Chuan-Li Lu [4], Hua Chang [5] and Xun Xiang [6,*]

[1] College of Science, Yunnan Agricultural University, Kunming 650201, China
[2] CAS Key Laboratory of Tropical Plant Resources and Sustainable Use, Xishuangbanna Tropical Botanical Garden, Chinese Academy of Sciences, Xishuangbanna 666303, China
[3] College of Food and Drug Engineering, Guangxi Vocational University of Agriculture, Nanning 530007, China
[4] Institute of Bioengineering, Guangdong Academy of Sciences, Guangzhou 510316, China
[5] College of Vetezrinary Medicine, Yunnan Agricultural University, Kunming 650201, China
[6] College of Animal Science and Technology, Yunnan agricultural University, Kunming 650201, China
* Correspondence: fanqingfeijilin@126.com (Q.-F.F.); xiangxun2000@163.com (X.X.)
† These authors contributed equally to this work.

Abstract: The development of Dai medicine is relatively slow, and *Zanthoxylum* has great economic and medicinal value. It is still difficult to obtain medicinal components from the low-polarity parts of *Zanthoxylum* belonging to Dai medicine. In this study, we introduced one simple and quick strategy of separating target compounds from the barks of *Z. acanthopodium* var. *timbor* by high-performance countercurrent chromatography (HPCCC) with an off-line anti-inflammatory activity screening mode. The development of this strategy was based on the TLC-based generally useful estimation of solvent systems (GUESS) method and HPCCC in combination. This paper presented a rapid method for obtaining target anti-inflammatory compounds. Three lignins were enriched by HPCCC with an off-line inhibition mode of nitric oxide production in lipopolysaccharide-stimulated RAW264.7 macrophage cells, using petroleum ether–ethyl acetate–methanol–water (3:2:3:2) as the solvent system. The results showed that this method was simple and practical and could be applied to trace the anti-inflammatory components of the low-polarity part in Dai medicine.

Keywords: *Zanthoxylum acanthopodium* var. *timbor*; anti-inflammatory activity screening; high-performance countercurrent chromatography; preparative separation

Citation: Fan, Q.-F.; Zhou, L.; Gongpan, P.-C.; Lu, C.-L.; Chang, H.; Xiang, X. Bioactivity-Guided High Performance Counter-Current Chroma-Tography and Following Semi-Preparative Liquid Chromatography Method for Rapid Isolation of Anti-Inflammatory Lignins from Dai Medicinal Plant, *Zanthoxylum acanthopodium* var. *timbor*. *Molecules* **2023**, *28*, 2592. https://doi.org/10.3390/molecules28062592

Academic Editors: Shihua Wu, Zhi Yang and Xinyi Huang

Received: 13 February 2023
Revised: 5 March 2023
Accepted: 9 March 2023
Published: 13 March 2023

Copyright: © 2023 by the authors. Licensee MDPI, Basel, Switzerland. This article is an open access article distributed under the terms and conditions of the Creative Commons Attribution (CC BY) license (https://creativecommons.org/licenses/by/4.0/).

1 Original spectra of isolated compounds 1-3

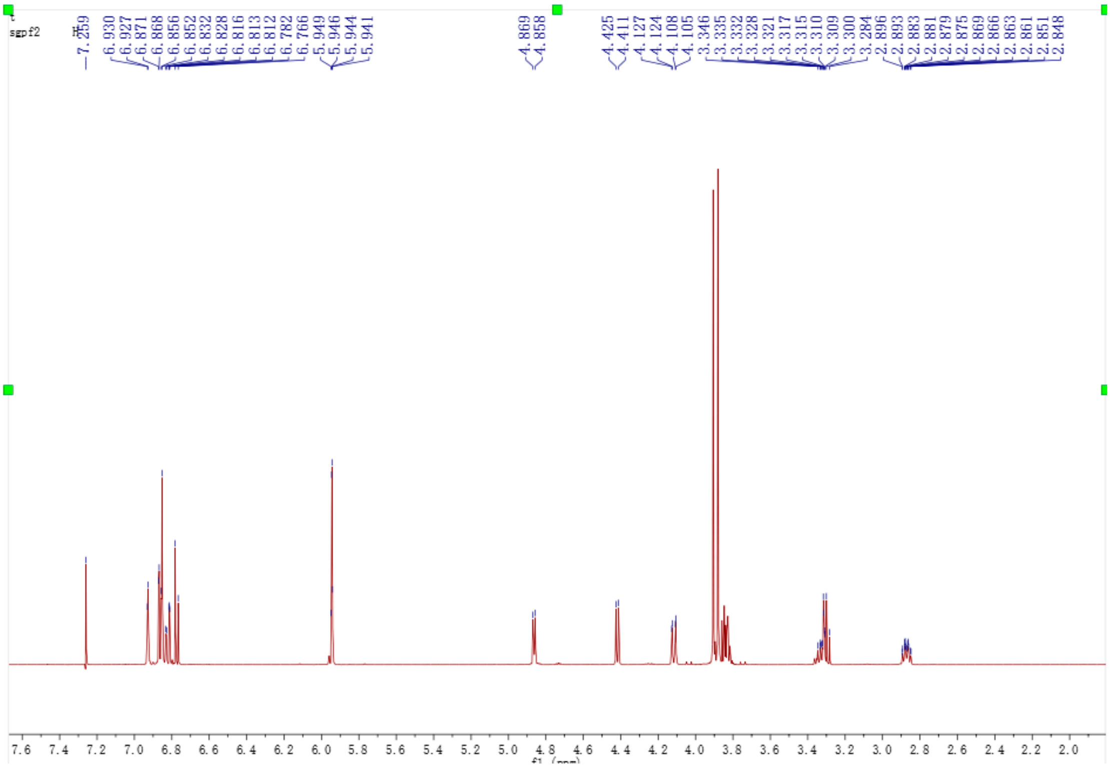

Figure S1. HNMR of compound **1.**

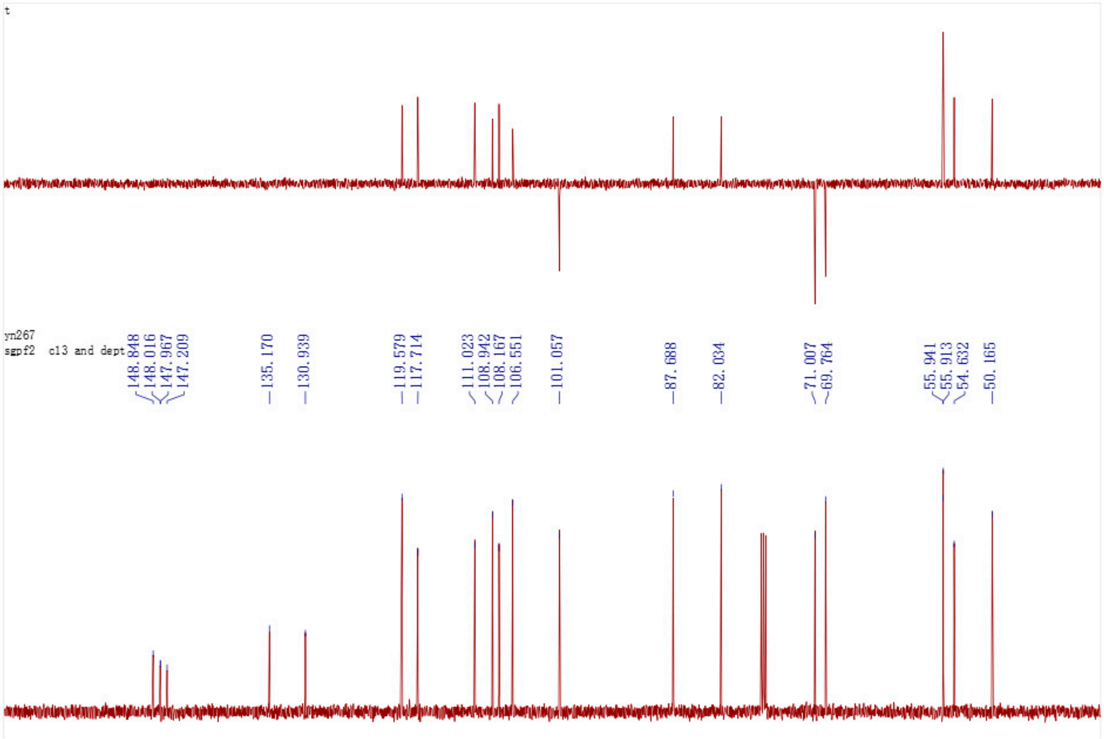

Figure S2. DEPT of compound **1.**

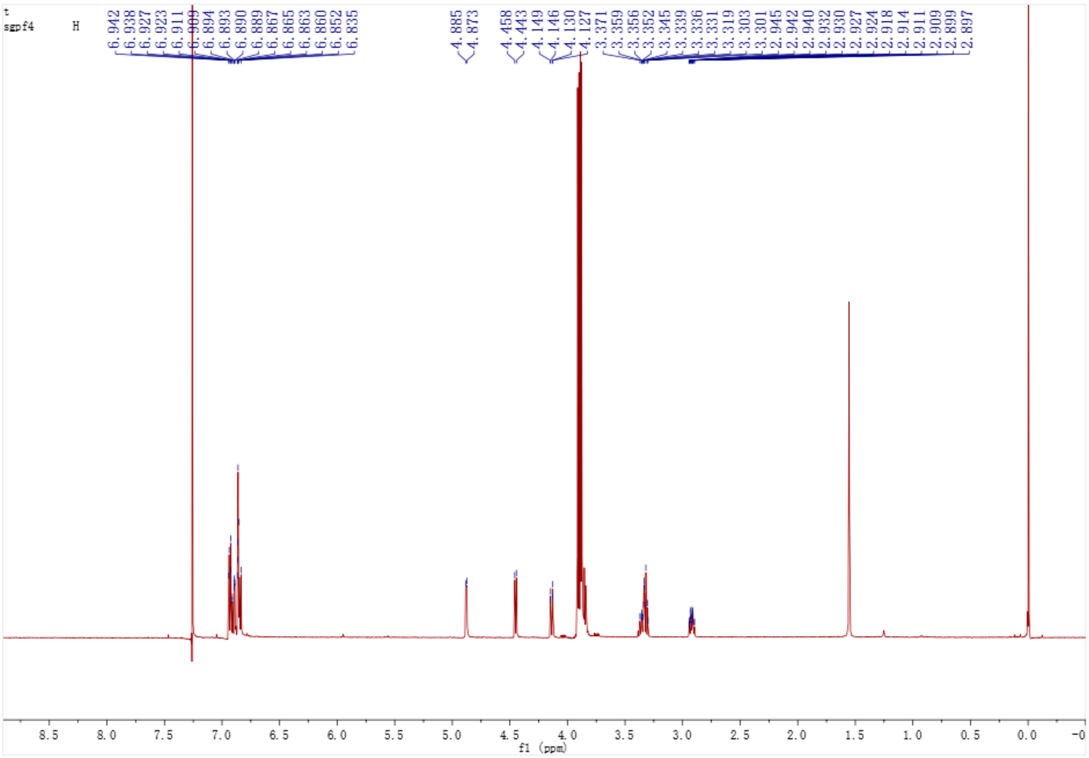

Figure S3. HNMR of compound **2.**

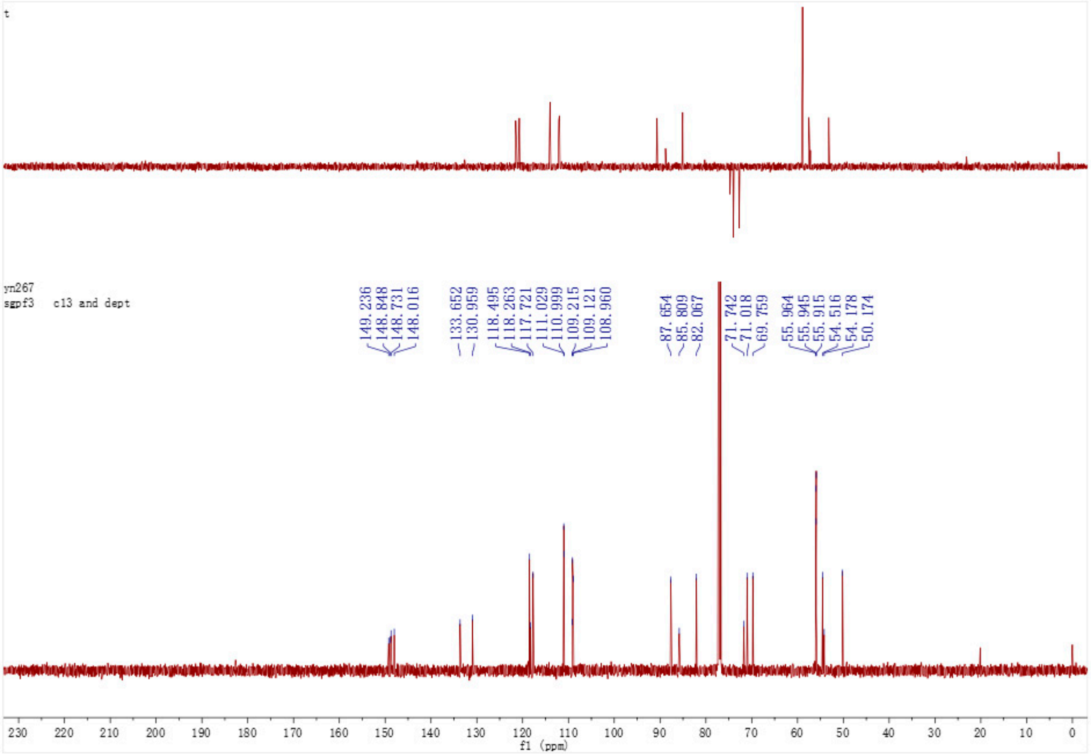

Figure S4. DEPT of compound **2.**

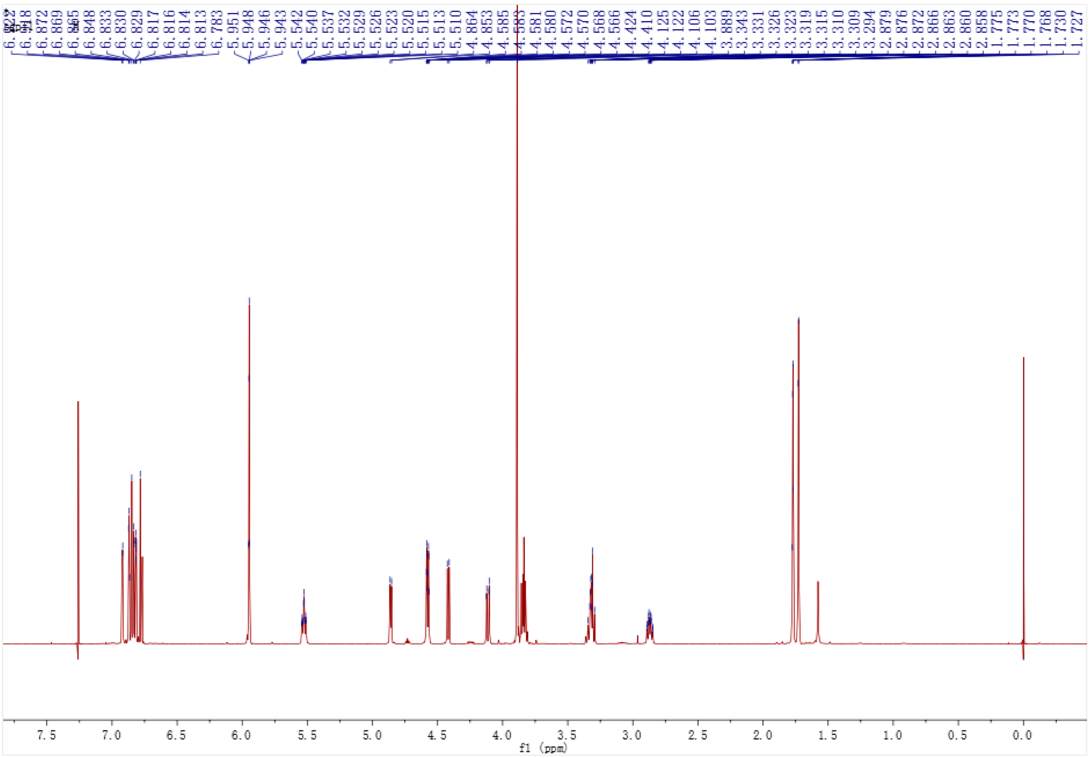

Figure S5. HNMR of compound **3.**

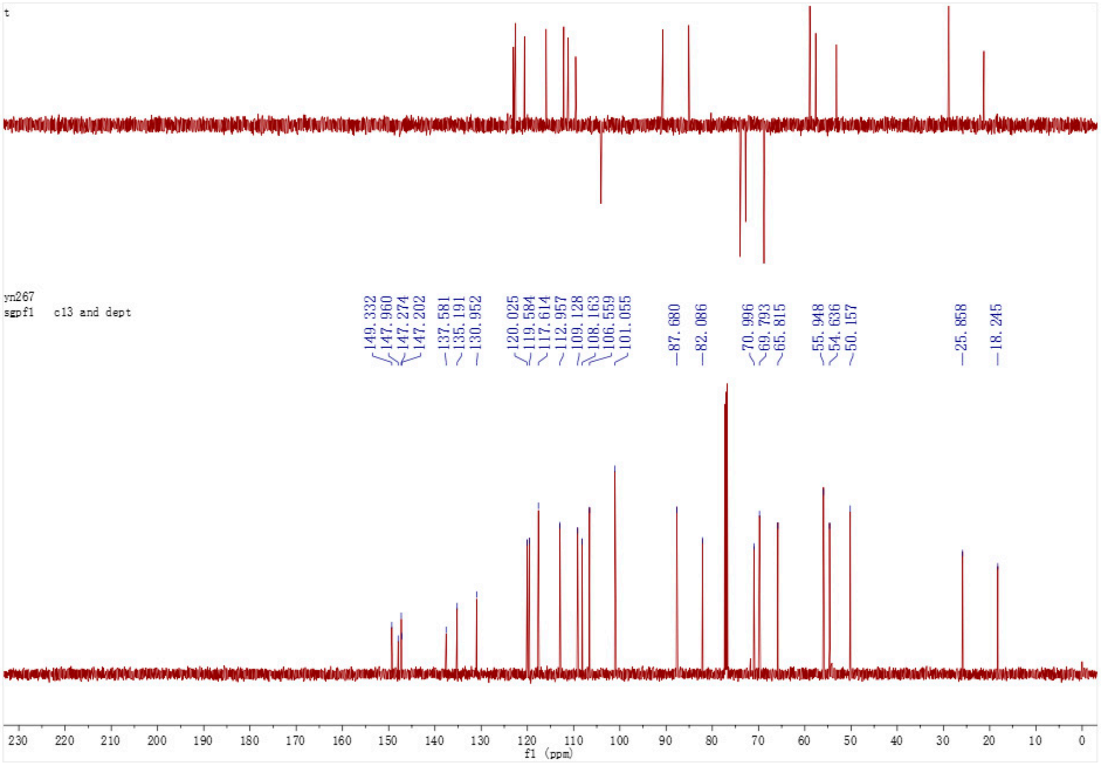

Figure S6. DEPT of compound **3.**

Disclaimer/Publisher's Note: The statements, opinions and data contained in all publications are solely those of the individual author(s) and contributor(s) and not of MDPI and/or the editor(s). MDPI and/or the editor(s) disclaim responsibility for any injury to people or property resulting from any ideas, methods, instructions or products referred to in the content.
